# Impact of the COVID-19 Outbreak on the Behavior of Families in Italy: A Focus on Children and Adolescents

**DOI:** 10.3389/fpubh.2021.608358

**Published:** 2021-02-05

**Authors:** Sara Uccella, Elisa De Grandis, Fabrizio De Carli, Maria D'Apruzzo, Laura Siri, Deborah Preiti, Sonia Di Profio, Serena Rebora, Paola Cimellaro, Alessandra Biolcati Rinaldi, Cristina Venturino, Paolo Petralia, Luca Antonio Ramenghi, Lino Nobili

**Affiliations:** ^1^Child Neuropsychiatry Unit, IRCCS Istituto Giannina Gaslini, Genoa, Italy; ^2^Department of Medical and Surgical Neuroscience and Rehabilitation (DINOGMI), University of Genoa, Genoa, Italy; ^3^Institute of Molecular Bioimaging and Physiology, National Research Council, Genoa, Italy; ^4^Psychology Unit, IRCCS Istituto Giannina Gaslini, Genoa, Italy; ^5^Health Care General Management Office, IRCCS Istituto Giannina Gaslini, Genoa, Italy; ^6^Neonatology Unit, IRCCS Istituto Giannina Gaslini, Genoa, Italy

**Keywords:** caregivers, behavioral changes, pandemic, stress, coping, psychological weaknesses, sleep, COVID–19

## Abstract

The COVID-19 pandemic has changed individuals' lifestyles to a great extent, particularly in Italy. Although many concerns about it have been highlighted, its impact on children and adolescents has scarcely been examined. The purpose of this study was to explore behavioral consequences and coping strategies related to the pandemic among families in Italy, by focusing on developmental ages from the caregivers' perspective, 3 weeks into quarantine. An exploratory cross-sectional online survey was conducted over 14 days. Google Forms was employed to conduct the survey. Demographic variables and pre-existing Psychological Weaknesses (PsW) were asked. Adults' sleep difficulties (SleepScore) and coping strategies during quarantine were assessed. Behavioral changes related to quarantine of both subjects completing the form (COVIDStress) and their children (when present) were questioned. Of the 6,871 respondents, we selected 6,800 valid questionnaires; 3,245 declared children aged under 18 years of age (caregivers). PsWs were recognizable in 64.9% among non-caregivers and in 61.5% of caregivers, with a mean PsW score of 1.42 ± 1.26 and 1.30 ± 1.25 over 3 points, respectively. The 95.5% of the non-caregivers and the 96.5% of caregivers presented behavioral changes with a mean COVIDStress of 3.85 ± 1.82 and 4.09 ± 1.79 over 8, respectively (*p*<0.001). Sleep difficulties were present in the 61.6% of the non-caregivers and in the 64.4% of the caregivers (*p* < 0.001), who showed higher SleepScores (2.41 ± 1.26 against 2.57 ± 1.38 points over 6, *p* < 0.001). COVIDStress (and SleepScore) strongly correlated with PsW (*p* < 0.001). Caregivers observed behavioral changes in their children in the 64.3% of the <6 years old and in 72.5% of 6–18 years old. Caregivers' discomfort related to quarantine (COVIDStress, SleepScore) was strongly associated to behavioral changes in both age groups of <6 and 6–18 (*p* < 0.001). Presence of caregivers' coping strategies was less associated to behavioral changes in the <6 sample (*p* = 0.001) but not in the 6–18 (*p* = 0.06). The COVID-19 pandemic has adversely impacted families in Italy with regard to behavioral changes, especially in high-risk categories with PsWs and caregivers, especially the ones with children aged <6 years. While coping strategies functioned as protective factors, a wide array of stress symptoms had implications for children's and adolescents' behaviors. It is recommended that public children welfare strategies be implemented, especially for higher-psychosocial-risk categories.

## Introduction

The coronavirus disease 2019 (COVID-19) pandemic (1) has rapidly changed countries' lifestyles. Numerous concerns about the long-term consequences of COVID-19 on the public health, economy, and sociality of the global human population have been sharply highlighted (2, 3). Italy was the first European country to experience the unpredictable consequences of the pandemic (4). Between March 1 and April 30, 2020, the average number of daily deaths recorded in Italy was 2,564. The majority of deaths occurred in the northern region, that is, Lombardy. To limit the rapid spread of the virus, a quarantine period was induced in Italy on March 9 (5).

Despite limiting the spread of infectious diseases, the state of quarantine may lead to the social isolation of families and individuals, causing or exacerbating psychological distress among the population (6–8). COVID-19, like other pandemic respiratory outbreaks (9–12), may be extremely pervasive and unforeseeable and may have a significant negative impact on mental health (8, 13).

Among studies on early behavioral changes in adults during COVID-19 quarantine, protective factors against psychological distress seem include older age, male gender, and physical health condition (14, 15). Moreover, it has been discussed that the confinement condition can lead to forced inactivity and increase in sedentary behavior, which are known to expose to increased risk for psycho-physical adverse conditions such as premature aging, obesity, cardiovascular vulnerability, muscle atrophy and bone loss (16) but also anxiety and depressive symptoms (17). Leading the quarantine to this change of habits, the importance of movement during the forced rest period of quarantine in the entire populations as a non-pharmacological and preventive treatment for the psychophysical well-being is very relevant (18, 19). A few studies have recently demonstrated the positive impact of home-based physical activity on adults in Italy (5, 18, 20). Moreover, sleeping regularly, eating healthy reducing the boredom and improve the communication by talking with friends and family members by either phone or socials have been considered good preventive coping strategy to mitigate consequences of quarantine (21). The World Health Organization, further suggested to minimize the exposure to news about the topic (21, 22).

The focus so far has been on the adult population, shedding light on the psychological impact among high-risk categories, such as healthcare and public workers (23). However, less attention has been given to the effects of this pandemic on children and adolescents (24). Our institute, the Gaslini Children's Hospital, created the “Families and Children cope with COVID-19” (Face-COVID-19) project during the early stages of the lockdown in Italy in early March. The purpose of this project was to support families and children during the critical phase of confinement. This was conducted through a dedicated anonymous phone and mail service that was managed by specialists in children and adolescents' psychopathology and trauma. Furthermore, the project also aimed to conduct an exploratory overview of behaviors and coping strategies during early phases of COVID-19 related quarantine among Italian families.

## Materials and Methods

Face-COVID-19 is an open project developed by the University of Genoa and the Child Neuropsychiatry and Psychology Units of the Gaslini Children's Hospital, Genoa, Italy. An online structured survey, the Face-COVID-19 Questionnaire, was developed and employed on Google Forms (https://docs.google.com/forms/d/e/1FAIpQLSfc3yjjn1az1hnVNt2BiDFYn6FdwTxe5dQhXf6b1y0rq17Fzw/closedform). It was administered to Italian families between March 23 and April 4. The survey was developed by two child and adolescent psychiatry specialists (SU and LN) and subsequently revised by five experienced psychologists (CV, MD'A, SD, DP, and SR).

The Local Ethics committee approved this study, according with the Declaration of Helsinki. The participating subjects provided online informed consent to collect data anonymously.

The questionnaire was officially released on the institutional website of the Gaslini Children's Hospital and disseminated through institutional and private social media, including Facebook, Instagram, WhatsApp, and Telegram. The online modality was chosen to respect social restrictions and confinement regulations. A temporal window of 14 days was considered appropriate for screening stress-related disturbances in adults and stress-related behavioral changes in children (25).

Voluntary participation was stimulated through the network in a critical phase of the epidemic to collect data from a large and motivated sample.

The inclusion criterion to participate in the survey was being of legal age, that is, over 18 years. *Post-hoc* analysis was focused on adults (caregivers) with children under 18 years, specifically those under 6 years and those between 6 and 18 years. The division in these two age groups follows criteria of American Psychiatry Association, described in the Diagnostic and Statistical Manual of Mental Disorders, 5th edition, for correctly identifying behavioral dangerous changes in the context of life-threatening events or major life-stressors (25). These criteria were also adopted in other investigations on this topic (26).

### The Families and Children Cope With COVID-19 Questionnaire (Face-COVID-19Q)

The Face-COVID-19 Questionnaire comprises 73 items. The focus of the first section is on adults: their pre-existing psychological fragilities, stress-related disturbances, and coping strategies are assessed. The second section is a parent-proxy report asking parents about their children and their behavioral changes. Furthermore, three questions examine a sense of loneliness, hopelessness, and a request for expert psychological aid. Geographical and demographic data are also collected. To address all socioeconomic categories, medical, and psychological terminology was avoided and simple and common terminology was used.

The complete questionnaire can be found in Appendix 1.

To afford an enhanced understanding of the data that were generated from the survey and to simplify them, a few referral scores were created (Appendix 2).

### Effective COVID-19 Threats: The COVIDThreat Score

A score derived from COVID-19 was attributed to each respondent who completed the survey by considering their answers to questions (items 13–17) about family members' and/or close friends' involvement in the pandemic, including testing positive, hospitalization and/or death, and chronic diseases with specific weekly treatments. The COVIDThreat score ranged from 0 to 5, with higher scores indicating more severe conditions.

### Pre-existence of Psychological Weaknesses in Adults: The PsW Score

Pre-existing PsWs from before the quarantine were assessed in questions 18, 19, and 20. The respondents were required to evaluate their pre-existing anxiety, depression, and sleep problems on a 4-point scale, ranging from 0 to 3.

### Sleep Habits During the Early Phases of the Quarantine in Adults: The SleepScore

This was a sleep-difficulties score calculated in the early phase of the outbreak, ranging from 0 to 6, determined by assessing questions 25–30.

### Substance Use by Adults During the Early Phases of the Quarantine: The SubUse Score

Substance use (consuming alcohol, smoking, and using drugs and psychopharmaceuticals) in larger quantities during the quarantine than before it or starting to use one or more of the substances because of the restrictive rules of confinement was explored in questions 48–51. The SubUse score ranges from 0 to 3.

### Symptoms Possibly Related to the COVID-19 Outbreak in Adults: The COVIDStress Score

To assess stress due to COVID-19, we developed a COVIDStress score that ranged from 0 to 8, with higher scores indicating more severe impairment. Questions 24, 38, 39, 41, 46, 71, 72, and 73 that investigated difficulties in concentrating; exacerbations of known chronic diseases such as allergic rhinitis, asthma, atopic dermatitis, itching, gastroesophageal reflux, constipation, diarrhea, and migraines; inexplicable physical sensations; fear of contamination; irritability; sense of loneliness; need for psychological help; and sense of hopelessness were employed to obtain the COVIDStress score.

### Coping Strategies of Adults: The CopingScore

Coping strategies, which are assessed in items 36, 37, and 44, comprise time spent engaged in sports, hobbies, and/or social interactions to obtain the CopingScore. As coping strategies are considered possible protective factors, the score ranges from 0 to −3.

### Children's and Adolescents' Behavioral Changes During Quarantine

Behavioral changes of children are assessed in two age groups: <6 years and 6–18 years.

The score is determined by summing the number of behavioral changes indicated by parents in questions 53 and 63, with a comprehensive score from 0 to 13 and from 0 to 9 in the respective age groups. Higher scores indicate more severe impairment.

### Statistical Analysis

Statistical analyses were performed by employing SPSS Statistics software, v23 (IBM, Armonk, NY, United States).

Exploratory statistical analysis was performed, taking into consideration the non-probability nature of the sample, based on unrestricted, self-selected survey (27). In order to estimate and compare expected values for different categorical and continuous variables and their association, the multiple comparison problem was considered, and the statistical significance threshold was set at a *p-*value of 0.001. Target sample size was estimated for the different parameters (expected scores, comparison of means and measure of association) at the desired level of significance (*p* < 0.001) and reasonably low effect size and a sample size > 2,500 was considered adequate (28).

Categorical variables are represented either as numbers and percentages in brackets or as medians with interquartile ranges. Continuous variables are listed with means and standard deviations. The Shapiro–Wilk test was performed to evaluate the distribution of the variables.

The chi-square test was employed to assess the association between categorical variables. Continuous variables were compared between groups by using unpaired *t-*test.

Spearman's correlation coefficients were used to determine the relationships between the scores representing different sets of behavioral disorders in parents and children.

Linear regression analysis was performed to investigate the reciprocal influences on the final result of psychic changes related to COVID-19 (COVIDStress) of the following specific categories: age, pre-existing PsWs, and presence of dependent children and/or of individuals older than 65 years of age.

Cronbach's alpha analysis was used to verify the validity and reliability of the questionnaire. For the validity analyses we considered questions assessing the malaise of the respondents and divided them in 3 sets: (1) those related to the adults' condition of discomfort and stress (items corresponding to COVIDThreat score, PsW score, COVIDStress score, and SleepScore); (2) those related to the discomfort of children aged under 6 years; (3) those related to the discomfort of children aged 6–18 years. We decided to not include the SubUse score and CopingScore questions as substance abuse and coping strategies can be considered as reaction to an adverse event and not only a feeling or a malaise (29).

## Results

**Figure 1:**
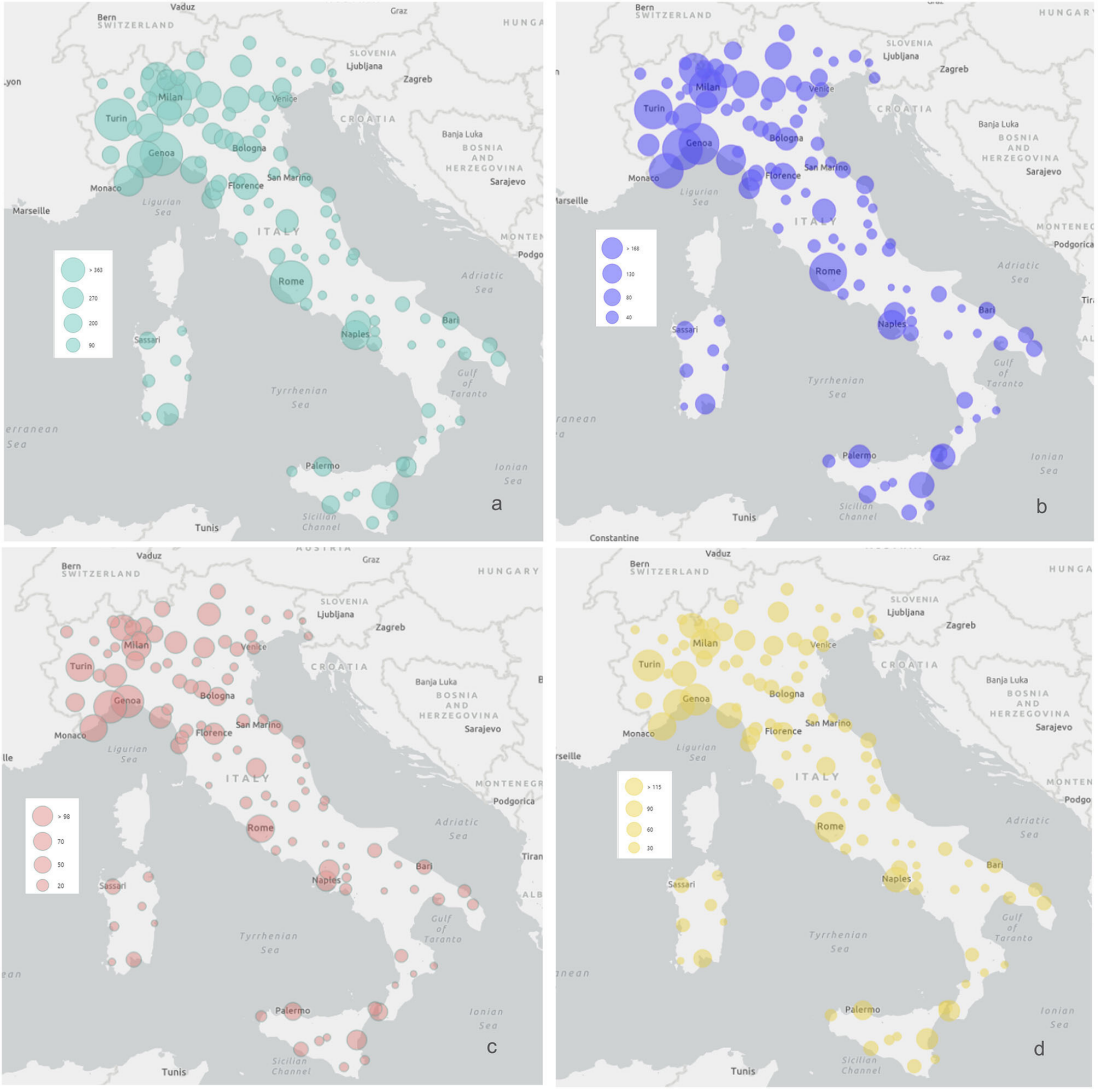
Geographical distribution of the sample. Geographical distribution of the number of respondents in each region. **(a)** Depicts the whole sample; **(b)** depicts the sample of 3,245 responding parents; **(c,d)** depict children aged <6 years and 6–18 years, respectively.

The questionnaire was completed by 6,871 respondents. Individual responses were then checked within the validity range and cross-checking was done to verify the consistency of responses, and therefore 71 questionnaires were excluded from the analyses. All the variables had a skewed distribution. The geographical distribution and demographic features of the sample are presented in Figure 1 and Supplementary Table 1.

Cronbach's alfa for questions regarding adults' malaise resulted 0.804. The ones regarding consistency of questions regarding behavioral changes in children aged under 6 years and over 6 years of age were, respectively 0.654 and 0.667.

### Effective COVIDThreat

On average, the respondents' score for COVIDThreat was 0.52 ± 0.77. The average score of caregivers and non-caregivers with people aged over 65 years among the households was significantly higher compared to the ones without (*p* < 0.0001). Also, caregivers with children with age <6 years had a higher COVIDThreat scores compared to caregivers with children with age 6–18 years (Table 1 and Supplementary Table 2).

**Table 1:** Summary of calculated scores among the whole sample and the subgroups of caregivers (subclusters <6 years and 6–18 years) and of individuals over 65 years of age.

	**COVIDThreat score Mean ± sds**	**COVIDThreat >1 n (%)**	**PsW score Mean ± sds**	**PsW > 1 ** **n (%)**	**SleepScore Mean ± sds**	**SleepScore > 1 n (%)**	**SubUse score Mean ± sds**	**SubUse score > 1 ** **n (%)**	**COVIDStress score Mean ± sds**	**COVIDStress Score > 1 ** **n (%)**	**CopingScore Mean ± sds**	**CopingScore > 1 n (%)**
Whole sample (*n =* 6,800)	0.52 ± 0.77	2,609 (38.4)	1.35 ± 1.26	4,251 (62.5)	2.57 ± 1.38	4,393 (64.6)	0.22 ± 50	1,240 (18.2)	3.87 ± 1.81	6,505 (95.6)	−1.10 ± 0.82	5,198 (76.4)
Caregivers with children aged <18 (*n =* 3,245)	0.54 ± 0.77	1,294 (39.9)	1.30 ± 1.25	1,996 (61.5)	2.71 ± 1.43	2,091 (64.4)	0.22 ± 0.49	595 (18.3)	4.09 ± 1.79	3,133 (96.5)	−1.01 ± 0.78	2,407 (74.2)
Non-caregivers (*n =* 2,336)	0.51 ± 0.78	879 (37.5)	1.42 ± 1.26	1,515 (64.9)	2.41 ± 1.37	1,440 (61.6)	0.24 ± 0.53	460 (19.7)	3.85 ± 1.82	2,331 (95.5)	−1.26 ± 0.87	1,891 (81.0)
*p*°	*0.143*	*0.089*	*** <0.0001***	*** <0.0001***	*** <0.0001***	*** <0.0001***	*0.146*	*0.201*	*** <0.0001***	*** <0.0001***	*** <0.0001***	*** <0.0001***
Caregivers with children aged <6 (*n =* 1,595)	*0.55 ± 0.77*	655 (41.1)	1.29 ± 1.24	978 (61.3)	2.82 ± 1.44	1,060 (66.5)	0.21 ± 0.49	293 (18.4)	4.31 ± 1.71	1,554 (97.4)	−0.98 ± 0.75	1,191 (74.7)
Caregivers with children aged 6–18 (*n =* 2,265)	0.53 ± 0.79	883 (39.0)	1.31 ± 1.25	1,398 (61.7)	2.66 ± 1.43	1,437 (63.4)	0.22 ± 0.50	429 (18.9)	4.02 ± 1.82	2,179 (96.2)	−1.0 ± 0.79	1,666 (73.6)
*p*°	*** <0.0001***	*** <0.0001***	*0.230*	*0.824*	*** <0.0001***	*0.058*	*0.537*	*0.685*	*** <0.0001***	*0.034*	*0.429*	*0.458*
Caregivers of children <18 years with people aged over 65 years among households (746)	0.67 ± 0.81	371 (49.7)	1.43 ± 1.25	498 (66.8)	2.86 ± 1.45	473 (63.4)	0.24 ± 0.51	151 (4.7)	4.12 ± 1.83	732 (98.1)	−0.97 ± 0.77	315 (73.3)
Caregivers of children <18 years without people aged over 65 years among households (2,499)	0.50 ± 0.76	923 (36.9)	1.26 ± 1.24	1,498 (59.9)	2.66 ± 1.41	1,618 (64.7)	0.20 ± 0.48	444 (17.8)	4.12 ± 1.80	2,401 (96.1)	−1.01 ± 0.79	903 (74.1)
*p*°	*** <0.0001***	*** <0.0001***	***0.001***	***0.001***	***0.001***	*0.502*	*0.187*	*0.125*	*0.195*	*0.007*	*0.55*	*0.739*
Non-caregivers with people aged over 65 years among households (858)	0.58 ± 0.83	361 (42.1)	1.48 ± 1.24	586 (68.3)	2.45 ± 1.35	533 (62.1)	0.25 ± 0.55	179 (20.9)	3.90 ± 1.85	823 (95.9)	−1.22 ± 0.86	687 (80.1)
Non-caregivers without people aged over 65 years among households (1,478)	0.47 ± 0.74	518 (35.0)	1.39 ± 1.27	929 (62.9)	2.38 ± 1.36	907 (61.4)	0.22 ± 0.51	281 (19.0)	3.81 ± 1.80	1,408 (95.3)	−1.27 ± 0.87	1,204 (81.5)
*p*°	*** <0.0001***	*** <0.0001***	*0.009*	*0.008*	*0.229*	*0.717*	*0.183*	*** <0.0001***	*0.249*	*0.534*	*0.178*	*0.409*

°statistical significance was calculated using the unpaired t-test to test differences between groups of continuous variables and chi-square test to test categorical variables. Significant values are highlighted in bold

### Pre-existence of Psychological Weaknesses in Adults

Various grades of pre-existing PsWs were recognizable in 62.5% of the sample, with a mean PsW of 1.35 ± 1.26. Caregivers showed lower PsW scores compared to non-caregivers respondents (*p* < 0.0001). Higher PsW scores were observed among caregivers with at least one person aged over 65 years of age among households (*p* < 0.0001) (Table 1).

### Sleep Habits During the Early Phases of the COVID-19 Quarantine in Adults

The average SleepScore of the sample was 2.57 ± 1.38. Caregivers, especially those who cared for children under the age of 6 years, had higher scores (*p* < 0.0001). Of interest, among caregivers, presence of people aged over 65 years of age was associated to higher Sleepscores (*p* < 0.0001) (Table 1 and Supplementary Table 3).

### Substance Use by Adults During the Early Phases of the Quarantine

More smoking and/or use of other substances during quarantine were reported by 18.2% of the whole sample, with no differences among caregivers and non-caregivers. Both groups had similar SubUse scores (Table 1).

### Symptoms Possibly Related to the COVID-19 Outbreak in Adults

The majority of the respondents, specifically 95.6% of the entire sample, suffered stress related to the COVID-19 outbreak. The sample's mean COVIDStress score was 3.87 ± 1.81. Caregivers had higher COVIDStress Score compared to the non-caergivers (4.09 ± 1.79 vs. 3.85 ± 1.82, *p* < 0.0001), with parents of children aged under 6 years of age scoring worse than the ones of children and adolescents (4.31 ± 1.71 and 4.02 ± 1.82, respectively, *p* < 0.001) (Table 1 and Supplementary Table 4).

### Coping Strategies of Adults

Coping strategies were employed by 76.4% of the whole sample. Caregivers employed less frequently coping strategies than non-caregivers (respectively, 74.2 and 81%, *p* < 0.001), with poorer CopingScores (−1.01 ± 0.78 vs. −1.26 ± 0.87, *p* < 0.001; Table 1).

### Impact of COVID-19 on Families in Italy

An examination of the age groups revealed that older respondents were less likely to suffer from pre-existing PsWs and COVIDStress. However, those in their late twenties and in their sixties had a higher SleepScore (Supplementary Table 5).

Spearman's correlation coefficient revealed that the respondents' COVIDStress correlated with PsW (*r* = 0.36; *p* < 0.0001), COVIDThreat (*r* = 0.122; *p* < 0.0001), SubUse (*r* = 0.15; *p* < 0.0001), and SleepScore (*r* = 0.49; *p* < 0.0001).

The sample's COVIDThreat correlated with the subjective sensation related to difficulties in falling asleep and restorative sleep, expressed on Likert scales (*r* = 0.53 and *r* = −0.07, respectively, *p* < 0.0001 for both).

The results of linear regression of the COVIDStress score on the PsW score, age of the respondents, dependent children, and respondents with people aged over 65 years among households is displayed in Table 2. All the factors had a significant effect (*p* < 0.0001) on COVIDStress.

**Table 2:** Linear regression model on the final results of psychological changes related to COVID-19 (COVIDStress) for specific categories (age, pre-existing psychological weaknesses, condition of being a Caregiver, and presence of people aged over 65 years of age among households).

	**Estimate**	**SE**	**tStat**	***p***
(Intercept)	3.326	0.07654	43.455	*0*
Age	−0.027089	0.0015508	−17.468	*** <0.0001***
PsW	0.56474	0.016305	34.636	*** <0.0001***
Caregivers	0.33483	0.038509	8.6947	*** <0.0001***
Presence people aged over 65 among households	0.2455	0.041722	5.8841	*** <0.0001***

*Number of observations: 6,800, Error degrees of freedom: 6,795*.

*Root Mean Squared Error: 1.55*.

*R-squared: 0.197, Adjusted R-Squared: 0.196*.

*F-statistic vs. constant model: 415*.

*SE stands for standard error and tStat stands for t stastistics. Significant values are highlighted in bold*.

### Impact of COVID-19 on Children and Adolescents

**Figure 2:**
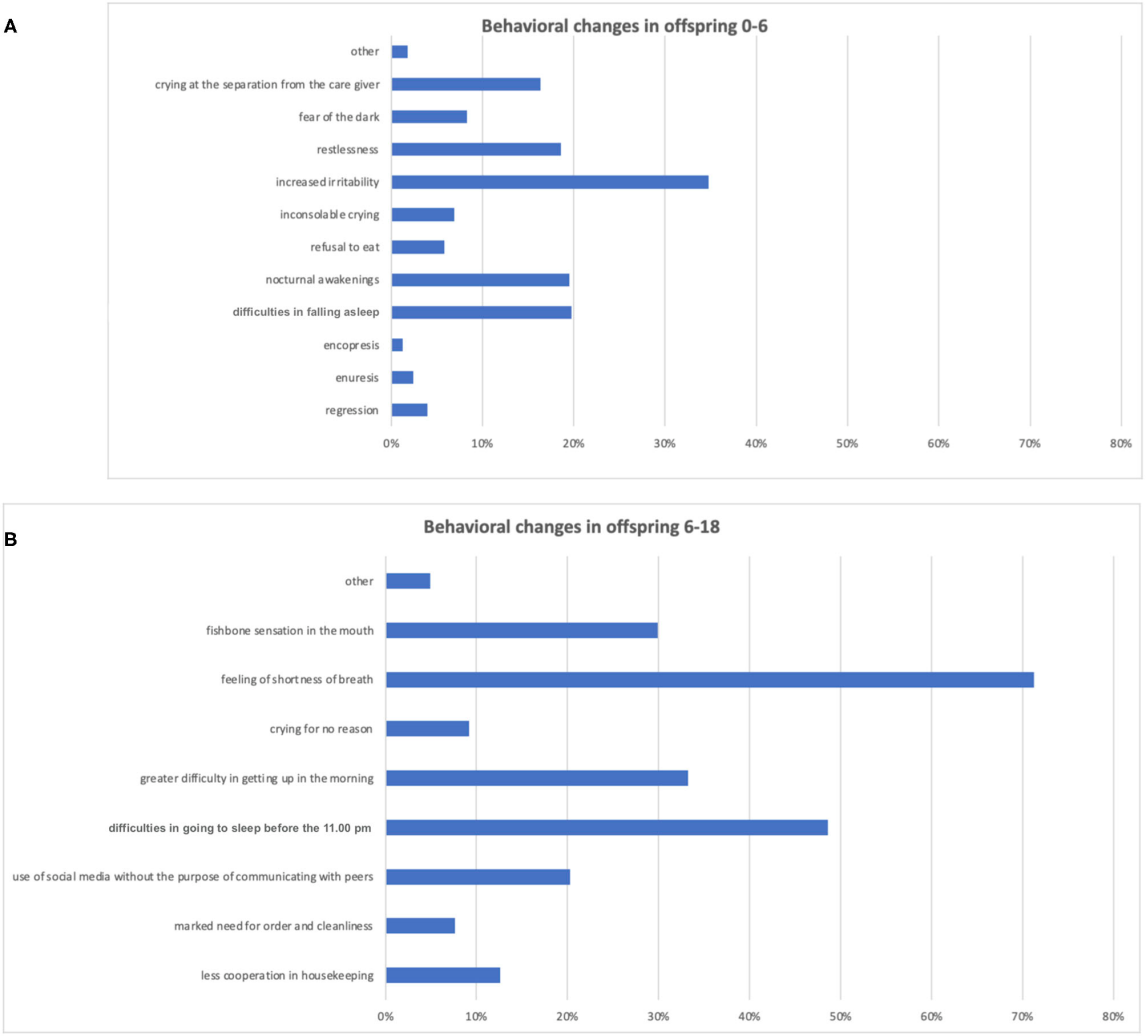
Behavioral changes reported by caregivers among children aged <6 years and aged 6–18 years. Istogram **(A)** depicts the distribution of behavioral changes among the group aged <6 years and istogram **(B)** of the group aged 6–18 years.

Behavioral changes in children were reported in 64.3 and 72.5% of the <6 years group and 6–18 years group, respectively. The average scores of both groups were 1.21 ± 1.39 and 2.39 ± 1.83, respectively (Figure 2).

Disorders that were frequently reported in the <6 years group included increased irritability (34.7%), sleep disorders, which constituted difficulties in falling asleep and night awakenings (19% each), and stress symptoms, such as restlessness (18.6%) and separation anxiety (16.4%). In the 6–18 years group, the main behavioral changes concerned somatic aspects, such as short breath (71.3%) as well as sleep problems, which comprised difficulties in falling asleep (48.6%) and waking up (33.2%).

Behavioral changes in both age groups were strongly (*p* < 0.0001) associated with the degree of parental discomfort following the COVID-19 outbreak (SleepScore, SubUse, and COVIDStress) and also with pre-existing PsWs (Table 3 and Supplementary Table 6).

**Table 3:** Spearman's correlations among psychological weaknesses and COVID-19-related behavioral disturbances among the caregivers with children in the age groups of <6 years and 6–18 years.

**Caregivers with children aged <6 (*n =* 1,595)**					
	**PsW**	**COVIDStress**	**SubUse**	**SleepScore**	**Children's behavioral changes**
PsW	1.00				
COVIDStress	0.38^*^	1.00			
SubUse	0.14^*^	0.13^*^	1.00		
SleepScore	0.40^*^	0.50^*^	0.13^*^	1.00	
Children's behavioral changes	0.20^*^	0.31^*^	0.07+	0.28^*^	1.00
**Caregivers with children aged 6–18 (*****n=*** **2,265)**					
	**PsW**	**COVIDStress**	**SubUse**	**SleepScore**	**Children's and adolescents' behavioral changes**
PsW	1.00				
COVIDStress	0.40^*^	1.00			
SubUse	0.13^*^	0.17^*^	1.00		
SleepScore	0.42^*^	0.49^*^	0.11^*^	1.00	
Children's and adolescents' behavioral changes	0.23^*^	0.36^*^	0.13^*^	0.23^*^	1.00

*Only signitificant correlations have been reported*.

*Nominal significance of the correlation coefficient (without correction for multiple tests)*.

* *p < 0.0001*,

+ *p < 0.001*.

Fewer behavioral changes in the children were associated with their caregivers' put in act of coping strategies (CopingScore) in the <6 years group (*r* = 0.077; *p* = 0.001) but not in the 6–18 years group (*r* = 0.009; *p* = 0.06).

## Discussion

Our results revealed that the COVID-19 pandemic outbreak affected people living in Italy early and dramatically in terms of unfavorable behavioral changes. The majority of the adult respondents (95.6%) were somewhat affected (COVIDStress) in face of the direct experience of COVID-19 (COVIDThreat). The latter was particularly prevalent among families with younger dependent children and among respondents with pre-existing PsWs. Moreover, our findings also highlighted the negative impact of the COVID-19 pandemic outbreak on children. Behavioral changes related to COVID-19 were found in almost two-thirds of the sample. A strong correlation was also revealed between caregivers' discomfort due to COVID-19 and their children's malaise.

COVID-19 has altered countries' health policies and lifestyles rapidly. Furthermore, the pandemic has had a severe impact on the general population, especially on those in high-risk categories (7, 30).

Although children appeared to have been less affected than adults from a medical perspective (18), their well-being has been at high risk: first, they faced the possibility of losing or being separated from their parents; second, they suffered a lack of social interactions, which was due to the closure of schools and kindergartens; third, family incomes may have been reduced; and fourth, their risk to the exposure of direct and indirect domestic violence may have been exacerbated (31–33), with a major risk for child neglect and abuse (34). The role of families has been highlighted as either a protective or aggravating factor during pandemics (6). However, the latter has not been examined adequately in scientific reports on the COVID-19 outbreak (24).

A parent-proxy survey conducted in China regarding children's discomforts during the second week of the COVID-19 quarantine revealed that children displayed high degrees of irritability. While children <6 years of age tended to manifest the fear of losing their caregivers, inattention was the most common manifestation in the 6–18 years group. They concluded that parents have a fundamental role in recognizing and managing their children's negative emotions timeously. However, relationships between children's and caregivers' well-being were not analyzed (26).

The primary aim of our study was to investigate such a possible link.

Most respondents were women in their early forties. This concurs with other surveys conducted on the psychological impact of the COVID-19 outbreak in Italy (35). This could be linked to the fact that women 30–50 years of age tend to use social media more, including Facebook and WhatsApp, through which the survey was disseminated. Furthermore, mothers were more involved in the stress related to the quarantine conditions, especially in relation to children younger than 6 years due to work and childcare issues (32).

The adult respondents reported mild to moderate levels of COVIDStress on average, even though there was a wide range among the sample. Our results are similar to those of Wang et al. (15) who conducted research at the start of the Chinese lockdown.

Stress related to COVID-19 (3.87 ± 1.81) correlated significantly with pre-existing PsWs, which were present in more than half of the respondents. This concurs with Moccia et al. (35) who revealed the high impact of the COVID-19 pandemic on individuals with PsWs, especially among those who are cyclothymic, depressive-anxious, and/or suffer insecure attachment.

Furthermore, we found a greater state of malaise among respondents who had dependent children in the <6 years group. Moreover, they suffered higher discomfort related to COVID-19 (COVIDStress), sleep problems related to COVID-19 (SleepScore), and fewer coping skills (CopingScore) (Table 1), thus revealing high levels of distress in this group.

Children and adolescents appeared to be less affected by the COVID-19 outbreak than their parents, even though they experienced considerable discomfort.

A recent review concerning the effects of COVID-19 related quarantine on mental health of children highlighted that young children have dealt with higher stress, showing higher levels of clinginess, sleep disturbances, nightmares, poor appetite, inattentiveness, and significant separation problems (36). More in detail, in a survey involving children and pre-adolescents in Hubei, Xie et al. revealed that 22.6 and 18.9% of the participants suffered depressive and anxiety symptoms, respectively, a month after the start of the Chinese quarantine. These results were not associated with demographic characteristics (38).

In our study adolescents seem less affected than younger children category. Buzzi et al. revealed that 65.7% of teenagers were moderately–severely worried about the COVID-19 pandemic but less than their parents (37). Oosterhoff studied the psychosocial implications of the COVID-19 outbreak among adolescents who, even when engaged in preventing the spread of the virus, needed to share experiences and deal with their experience of quarantine and social isolation (39). By the way, an early literature review has pointed out the insufferable nature of the COVID-19 related quarantine for some adolescents, who, depending also on their familiar socio-economic and mental condition (36), can experience a so-called *social craving*, with neural craving responses similar to neurofunctional circuits of hunger (40) and more discreet symptoms than the youngsters, such as sleep disturbances, problems with peers, isolation, and depression (41).

In this study, behavioral problems were present in 65 and 71% of children under and over the age of 6 years, respectively. Children under the age of 6 years frequently displayed increased irritability, sleep disorders, and anxiety problems (Figure 2A). This is in accordance with Jiao et al. (26).

Those with children over the age of 6 years experienced somatic complaints, such as the feeling of shortness of breath. Furthermore, they suffered significant alterations in sleep, particularly the inability to fall asleep as well as increased emotional instability, irritability, and changes in mood.

The questionnaire had not been previously validated as it was developed to catch behaviors and changes associated with the emergency situation without the adoption of previously standardized scales. This may limit comparisons with other studies but enables the evaluation of pandemic-related features, with an approach similar to that adopted in previous studies (8). In order to evaluate the reliability of the questionnaire and relevant scores we computed Cronbach's alpha for the sets of items related to the condition of discomfort and stress. For the set of items relevant to respondent condition, Cronbach's alpha was 0.804, indicating a good internal consistency, that is selected items provided a coherent indication of the level of malaise. When items relevant to children under or over 6 years of age were considered, Cronbach values were 0.654 and 0.667, respectively, which can be considered low borderline values of internal consistency reliability (42). This suggests multidimensionality, namely behaviors and reaction to pandemic could be more diversified in children, with different manifestations of malaise.

Our study demonstrated a strong relationship between the level of severity of children's dysfunctional behavior and the degree of the circumstantial malaise of their parents. This correlation was significantly accentuated in the case of prior psychological problems in caregivers (7, 32).

When applied, coping strategies among caregivers have acted as protective factors among the children (43).

The data need a diachronic perspective so as to be interpreted correctly.

The generalizability of our findings may be limited, as this study analyzes a non-probability sample, and the survey was open to participation of interested people. This can bring about a bias in the sample composition and consequently in the estimation of parameters not directly ascribable to the general population. The large and varied sample could however reveal remarkable trends and show differences between subgroups and associations between variables. Moreover, although the online survey allowed respondents throughout the country to respond, the number of respondents who had children under the age of 6 years was not the same as the number with children over the age of 6 years. A potential bias of selection may have resulted from the fact that those who suffer the most are more inclined to complete questionnaires. The results may have differed if children and adolescents had also answered the questionnaire. However, to avoid asking potentially overly emotional questions to them (for example, regarding the death of relatives or parents), we decided not to include respondents under the age of 18 years.

Moreover, although we presented the socio-demographic characteristics of the sample in the Supplementary Material at Supplementary Table 1, we did not explore these characteristics in the final analysis, even though the sample was large and varied for the distribution of age, social position, region and other characteristics. By the way, these characteristics were not considered also in other similar studies on this topic or on other pandemics-related quarantine effects (8, 31, 36).

Another possible limitation is the fact that in our survey we did not investigate if the respondents had themselves get infected by COVID-19. Indeed, we did not want to make people expose too much on the topic that could have been (and be) discriminating (as the stigma of the disease could have prevented the completion of the whole survey). For the same reason we did add the option “I prefer not to answer” at all questions regarding the COVIDThreat score.

Finally, to ensure anonymity of the data, it was not possible to conduct a follow-up.

To the best of our knowledge, we are the first to investigate the early impact of the COVID-19 quarantine in Italy.

The high number of responses to our questionnaire in a brief timeframe of 14 days highlights the population's high interest in the challenges of the pandemic.

The timeframe of the survey allowed for the onset of stress symptoms (25).

Furthermore, the double perspective of the caregivers on themselves and their children stressed the importance of the parental dyad, which, although fundamental for the well-being of children, has not been formally explored in previous studies (6, 8, 44).

## Conclusions

In conclusion, with our study, although limited by sample selection biases and the avoidance of standardized previously published scales, we have been able to show early malaise among people living in Italy due to COVID-19 related quarantine. The discomfort was highly accentuated in people suffering from mental fragilities (8) but also among participants to the survey without previous declared antecedents of psychological weaknesses but caregivers of children under 6 years of age. Moreover, the level of severity of children's dysfunctional behavior and the degree of the circumstantial malaise of their parents were strongly associated.

Efforts related to children's welfare strategies and addressing higher psychosocial risk categories should be implemented in the following months to maintain children's and adolescents' mental health.

## Data Availability Statement

The raw data supporting the conclusions of this article will be made available by the authors, without undue reservation.

## Ethics Statement

The studies involving human participants were reviewed and approved by Comitato Etico Regione Liguria. The patients/participants provided their written informed consent to participate in this study.

## Author Contributions

SU designed the study, constructed the databases, interpreted the data, and drafted the manuscript. LN, FD, and LR interpreted the data and revised the manuscript. FD made the statistical analysis. CV, MD'A, SP, DP, LS, ED, SR, AR, and PP helped in revising the final questionnaire. All authors helped in data collection, revised the manuscript, discussed the results, and commented on the manuscript.

## Conflict of Interest

The authors declare that the research was conducted in the absence of any commercial or financial relationships that could be construed as a potential conflict of interest.
