# Supplementary material for: Impact of the COVID-19 Outbreak on the Behavior of Families in Italy: A Focus on Children and Adolescents
Source: Front Public Health. 2021 Feb 5;9:608358. doi: 10.3389/fpubh.2021.608358 (PMC7893111; doi:10.3389/fpubh.2021.608358)
Supplement: Supplementary file 1 [file Data_Sheet_1.docx]

**Appendix 1 – Families and Children cope with COVID-19 Questionnaire (Face-COVID-19 Questionnaire)**

| *This questionnaire is addressed to all Italians over 18 years of age and was developed with the aim of understanding the impact that the recent COVID-19 pandemic has had on Italian families.*  *Your answers are important to enable a complete understanding of the situation so as to find the right intervention strategies to provide support in such a delicate moment that involves everyone.*  *This investigation is promoted by the IRCCS Giannina Gaslini Hospital.*  *We wish to inform you that this questionnaire is anonymous and uses a third-party tool (privacy information of the third-party service is available here:* [*https://policies.google.com/privacy)*](https://policies.google.com/privacy))*. The data collected with this tool by Istituto Giannina Gaslini will be treated in compliance with the privacy law Regulation (EU) 216/679.*  *To view Istituto Giannina Gaslini’s complete privacy policy, click here:* [*http://www.gaslini.org/privacy*](http://www.gaslini.org/privacy) | |
| --- | --- |
| **Question** | **Answer** |
| ***Section 1a*** | |
| 1. What is your gender? | female/male |
| 1. How old are you? | Answer in years (>18) |
| 1. In which province do you live? |  |
| 1. Level of education | primary school diploma  secondary school diploma  high school diploma  bachelor's degree  master’s degree or single cycle degree  other higher education |
| 1. What kind of work do you do? | Administrative-Employee/Craftsman-Maneuver/Police Forces/Freelancer/Factory worker/Healthcare Professional/Restauranteur/Student/Other/Unemployed |
| 1. How many people does your family comprise of? | 1/2/3/4/5/more than 5 |
| 1. Do you have any children? | yes/no |
| 1. How many of your children are dependent on you? | 0/1/2/3/4/more than 4 |
| 1. Of these, how many are younger than 6 years? | 0/1/2/3/4/more than 4 |
| 1. How many are between the ages of 6 and 18 years? | 0/1/2/3/4/more than 4 |
| 1. How many people aged over 65 years are there in your family? | 0/1/2/3/4/more than 4 |
| 1. How would you rate the impact that the current COVID-19 pandemic could have on the global population? | Likert scale from 0 to 10 (0: not important at all; 10: extremely important) |
| 1. Has anyone in your family tested positive for COVID-19? | yes/ no/I don't know/I prefer not to answer |
| 1. Have any of your family members or close circle of friends been admitted to the hospital because of COVID-19? | yes/no/I prefer not to answer |
| 1. Have any of your family members died of COVID-19? | yes/no |
| 1. Has anyone in your close circle of friends died of COVID-19? | yes/no |
| 1. Do any of your family members need specific weekly treatments because of chronic illnesses? | yes/no |
| 1. Have you ever had an anxiety problem, even one not diagnosed by a specialist? | yes/no/I don’t know |
| 1. Have you ever had problems with depression or marked sadness, even if not diagnosed by a specialist? | yes/no/I don’t know |
| 1. Have you ever had problems with sleep? | yes/no |
| ***Section 1b***  *Please respond with regard to the past few weeks, starting Monday, March 9.* | |
| 1. Since the introduction of more restrictive measures to prevent contagion, my number of weekly working or study hours… | has remained the same/increased/decreased/I no longer work |
| 1. Are you still in contact with the public or unknown people during the day due to work activities? | yes/no |
| 1. Have you started using a smart-working mode for working? | yes/no |
| 1. From the introduction of more restrictive measures of contagion prevention, concentrating … | is the same/has become easier/has become more difficult |
| 1. From the introduction of more restrictive measures of contagion prevention, my number of sleeping hours … | has not changed/ has increased/has decreased |
| 1. Currently, for how many hours do you sleep each night? | Less than 5 hours/5/6/7/8/9/more than 9 hours |
| 1. On a scale of 1–10, rate how hard it is for you to fall asleep. | Likert scale from 1 (very easy) to 10 (very hard) |
| 1. Do you have nocturnal awakenings? | yes/no |
| 1. Do you have nightmares? | yes/no |
| 1. How rested do you feel in the morning after sleeping? | from 1(little rested) to 10 (very rested) |
| 1. Have your eating habits changed? | They have remained unchanged/I’m hungrier, and I eat more/I’m less hungry, and I eat less |
| 1. Compared to three weeks ago, do you eat healthier? | yes/no |
| 1. Compared to three weeks ago, have you noticed that you have increased your intake of sweets and carbohydrates? | yes/no |
| 1. On a scale of 1–10, rate how many times you get hungry during the day (both before and after eating)? | Likert scale from 1 (I’m never hungry, in fact I often prefer not to eat) to 10 (10 or more times during the day) |
| 1. Are you, at times, unable to stop eating even when you feel full? | yes/no |
| 1. The time I spend on my hobbies… | has remained the same/I spend lesser time on my hobbies/I spend more time on my hobbies |
| 1. I play sports… | the same as before/lesser than before/more than before |
| 1. Have any of these chronic conditions, which you may suffer from, worsened? | allergic rhinitis/asthma/atopic dermatitis/itching/ gastroesophageal reflux/constipation/ diarrhea/migraine/other/none |
| 1. Have you experienced physical sensations that you can’t explain (palpitations, chest pain, sore throat, feelings of extraneousness or something else)? | yes/no |
| 1. Rate how many physical sensations that you are unable to explain (palpitations, chest pain, sore throat, headache, feelings of extraneousness or other) happen to you during the day. | Likert scale from 0 (none) to 10 (more than 10 per day) |
| 1. Are you more irritable or do you have sudden mood swings? | yes/no |
| 1. If you suffer from sudden mood swings, how many times a day does this occur? | Likert scale from 0 (never) to 10 (more than 10 times a day) |
| 1. Your physical contact with the people living with you… | has remained the same/decreased/increased |
| 1. Your phone contact or video calls with your friends… | have remained the same/decreased/increased |
| 1. How much time a day do you spend on the phone or social networks in a way that is NOT functional (having contact with people, working or getting informed)? | Likert scale from 0 (I don’t use social media for entertainment or play) to 10 (I spend 10 hours or more on social media for entertainment or play) |
| 1. On a scale of 0–10, rate how long you spend tidying up and cleaning the house or environment where you spend most of your time. | Likert scale from 0 (I can’t and don’t feel like spending time tidying up and cleaning) to 10 (I realize I spend most of my time tidying up and cleaning). |
| 1. The cleaning of house spaces is due to the need to control the dirt in the house for fear that it is contaminated by viruses or bacteria. | Likert scale from 0 (not at all) to 10 (very much) |
| 1. Do you drink alcohol? | yes, and I have had more than I did three weeks ago/ yes, but I drink the same amount as three weeks ago/yes, and I wasn’t used to drinking three weeks ago/no, but I drank three weeks ago/no, and I didn’t drink it until three weeks ago |
| 1. ..about smoking | yes, and I didn’t smoke until three weeks ago/yes, but I was smoking three weeks ago too/yes, and I smoke more than I used to three weeks ago/no, but I used to smoke three weeks ago/no, and I didn’t smoke three weeks ago |
| 1. ..about substance use | yes, and I have used more than I did three weeks ago /yes, but I used the same amount as three weeks ago/yes, and I didn’t use until three weeks ago/no, but I used three weeks ago/no, and I did not three weeks ago |
| 1. ..about psychotropic drugs | yes, and I have taken more of the drugs than I did three weeks ago / yes, but I took the same amount as three weeks ago/yes, and I didn’t take until three weeks ago/no, but I took it three weeks ago/no, and I did not three weeks ago |
| 1. On a scale of 0–10, rate how long you spend talking or reading about the COVID-19 epidemic during a day on average. | Likert scale from 0 (I prefer not to get informed and avoid the subject) to 10 (very frequently, at least every hour) |
| ***Section 2***  *Please respond with regard to the past few weeks, starting Monday, March 9* | |
| 1. If you have dependent children under the age of 6, have you observed any of these behaviors? | I don't have dependent children under the age of six/loss of linguistic skills/he/she started wetting the bed again/he or she soils his/her pants/difficulty falling asleep/nocturnal awakenings/refusal to eat/inconsolable crying/increased irritability/restlessness/fear of the dark/crying on being separated from you/other |
| 1. If you have dependent children under the age of 6, on a scale of 0–10, rate how many times a day they are exposed to news/images about COVID-19. | Likert scale from 0 (never) to 10 (always)/ I don’t have dependent children under the age of 6 |
| 1. If you have dependent children under the age of 6, do they ask about COVID-19? | yes/no/I don’t have dependent children under the age of 6 |
| 1. I If you have dependent children under the age of 6 who ask questions or talk about COVID-19, on a scale of 0–10, rate how often they speak about it. | Likert scale from 0 (never) to 10 (always)/I don’t have dependent children under the age of 6 |
| 1. If you have dependent children under the age of 6, have they expressed fear about the future? | yes/no/I don't have dependent children under the age of six |
| 1. If you have dependent children under the age of 6, have they been asking questions about the future? | yes/no/I don't have dependent children under the age of six |
| 1. If you have dependent children under the age 6, have they been asking more questions about death? | yes/no/I don't have dependent children under the age of six |
| 1. If you have dependent children under the age of 6, have they ever told you that they are afraid of losing their grandparents or parents? | yes/no/I don't have dependent children under the age of six |
| 1. If you have dependent children under the age of 6, have they started feeling afraid to go to sleep alone? | yes/yes but they also did it before /no/I don't have dependent children under the age of six |
| 1. If you have dependent offspring under the age 6 who need weekly physical/psychological rehabilitative treatment, how have the treatments continued? | We had to stop all treatments, and we can't do anything at home/we had to stop all treatments, but my spouse and I have continued the treatment at home/we have started online treatments with therapists/we have started online treatments with the operators, and we are applying the therapists’ instructions at home on our own/I don’t have any dependent children under the age of 6 who need this kind of intervention |
| 1. If you have dependent children over the age of 6, have you observed any of these behaviors? | I don’t have dependent children over the age of 6/less cooperation in housekeeping/marked need for order and cleanliness/use of social media without the purpose of communicating with peers/difficulty falling asleep before 23:00/greater difficulty in waking up in the morning/crying for no reason/feeling shortness of breath/fishbone sensation in the mouth/other |
| 1. If you have dependent children over the age of 6, on a scale of 0–10 rate how many times a day they are exposed to news/images about COVID-19. | Likert scale from 0 (never) to 10 (always)/I don’t have dependent children over the age of 6 |
| 1. If you have dependent children over the age of 6, do they ask about COVID-19? | yes/no/I don’t have dependent children over the age of 6 |
| 1. If you have dependent children over the age of 6 who ask questions or talk about COVID-19, on a scale of 0–10, rate how often they speak about it. | Likert scale from 0 (never) to 10 (always) |
| 1. If you have dependent children over the age of 6, have your children expressed fear about the future? | yes/no/I don’t have dependent children over the age of 6 |
| 1. If you have dependent children over the age of 6, have they expressed fear about death? | yes/no/I don’t have dependent children over the age of six |
| 1. If you have dependent children over the age of 6, have any of them told you that they are afraid of losing their grandparents or parents? | yes/no/I don’t have dependent children over the age of six |
| 1. If you have dependent children over the age of 6 who need weekly physical/psychological rehabilitative treatment, how have the treatments continued? | We had to stop all treatments, and we can’t do anything at home/we had to stop all treatments, but my spouse and I have continued the treatments at home/we have started online treatments with therapists/we have started online treatments with the operators, and we are applying the therapists’ instructions at home on our own/I don’t have any dependent children over the age of six who need this kind of intervention. |
| 1. Do you feel lonelier in comparison to the last two weeks? | yes/no |
| 1. On a scale of 0–10, how confident are you about the future? | Likert scale from 0 (null) to 10 (has increased a lot) |
| 1. Do you feel the need for specialized help right now? | yes/no |
| *Thank you for completing this survey*. | |

*Appendix 2* – Scores constructions

|  | Score name | Items | Question | Scoring | Grading |
| --- | --- | --- | --- | --- | --- |
|  | COVIDThreat | **13** | Have any of your family members tested positive for COVID-19? | **1 point if the answer is “yes”** | **0–5** |
|  |  | **14** | Have any of your family members or close of friends been admitted to the hospital because of COVID-19? | **1 point if the answer is “yes”** |  |
|  |  | **15** | Have any of your family members died of COVID-19? | **1 point if the answer is “yes”** |  |
|  |  | **16** | Has anyone of your close circle of friends died of COVID-19? | **1 point if the answer is “yes”** |  |
|  |  | **17** | Do any of your family members need specific weekly treatments because of chronic illness? | **1 point if the answer is “yes”** |  |
|  | PsW | **18** | Have you ever had an anxiety problem, even one not diagnosed by a specialist? | **1 point if the answer is “yes” °** | **0–3** |
|  |  | **19** | Have you ever had problems with depression or marked sadness, even if not diagnosed by a specialist? | **1 point if the answer is “yes” °** |  |
|  |  | **20** | Have you ever had problems with sleep? | **1 point if the answer is “yes” °**  °The answer “I don’t know” is scored as 0. |  |
|  | SleepScore | **25** | From the introduction of more restrictive measures of contagion prevention, my number of sleeping hours… | **1 point if the answer indicates that sleep has decreased** | **0–6** |
|  |  | **26** | Currently for how many hours do you sleep each night? | **(10 – number of hours slept*) / 6**  *count 4 if respondent answered “less than 5 hours” and count 10 if respondent answered “more than 9 hours” |  |
|  |  | **27** | On a scale of 1–10, rate how hard it is for you to fall asleep. | **1 point if the answer is “yes”** |  |
|  |  | **28** | Do you have nocturnal awakenings? | **1 point if the answer is “yes”** |  |
|  |  | **29** | Do you have nightmares? | **1 point if the answer is “yes”** |  |
|  |  | **30** | How rested do you feel in the morning after sleeping? | **(10 – answers at the Likert scale) / 10** |  |
|  | SubUse | **48** | Do you drink alcohol? | **1 point if the answer indicates drinking more** | **0–4** |
|  |  | **49** | ..about smoking | **1 point if the answer indicates smoking more** |  |
|  |  | **50** | ..about substance use | **1 point if the answer indicates using more** |  |
|  |  | **51** | ..about psychotropic drugs | **1 point if the answer indicates taking more**  ^count 1 if subject started during quarantine |  |
|  | COVIDStress | **24** | From the introduction of more restrictive measures of contagion prevention, you find that concentrating is… | **1 point if the answer indicates that it is more difficult** | **0–8** |
|  |  | **38** | Have any of these chronic conditions, which you may suffer from, worsened? | **1 point if any of the listed diseases (including) other have been ticked** |  |
|  |  | **39** | Have you experienced physical sensations that you can’t explain (palpitations, chest pain, sore throat, feelings of extraneousness or something else)? | **1 point if the answer is “yes”** |  |
|  |  | **41** | Are you more irritable or do you have sudden mood swings? | **1 point if the answer is “yes”** |  |
|  |  | **46** | On a scale of 1–10, rate how long you spend tidying up and cleaning the house or environment where you spend most of your time. | **(answers^+^ at the Likert scale) / 10)** ^+^answers under 5 are considered equal to 0. |  |
|  |  | **71** | Do you feel lonelier in comparison to the last two weeks? | **1 point if the answer is “yes”** |  |
|  |  | **72** | On a scale of 0–10, how confident are you about the future? | **(10 – answers at the Likert scale) / 10** |  |
|  |  | **73** | Do you feel the need for specialized help right now? | **1 point if the answer is “yes”** |  |
|  | CopingScore | **36** | The time I spend on my hobbies… | **1 point if more time is spent on hobbies** | **0–(−3)** |
|  |  | **37** | I play sports… | **1 point if sports are played more than before** |  |
|  |  | **44** | Your phone contact or video calls with your friends… | **1 point if it has increased** |  |
|  | Behavioral changes in the <6 group | **53** | If you have dependent children under the age of 6, have you observed any of these behaviors? | **1 point for each of the problems (including other) that have been ticked** | **0–13** |
|  | Behavioral changes in the 6–18 group | **63** | If you have dependent children over the age of 6, have you observed any of these behaviors? | **1 point for each of the problems (including other) that have been ticked** | **0–9** |
