# Supplementary material for: Impact of the COVID-19 Outbreak on the Behavior of Families in Italy: A Focus on Children and Adolescents
Source: Front Public Health. 2021 Feb 5;9:608358. doi: 10.3389/fpubh.2021.608358 (PMC7893111; doi:10.3389/fpubh.2021.608358)
Supplement: Supplementary file 2 [file Data_Sheet_2.docx]

**Supplementary material**

*Supplementary Table 1 –* Demographic and sample characteristics.

|  | **Whole sample**  **(n = 6800)** | **Caregivers with children aged <18**  **(n = 3245)** | **Non-caregivers**  **(n = 2336)** | **Caregivers with children aged <6 years of age**  **(n = 1595)** | **Caregivers with children aged between 6-18 years of age**  **(n = 2265)** |
| --- | --- | --- | --- | --- | --- |
| Age in years  mean (sds)  median (IQR) | 43.5 ± 12.2  43 (18) | 42.4 ± 7.5  42 (11) | 36.9 ± 12.0  36 (17) | 37.8 ± 5.64  37 (7) | 44.8 ± 6.99  45 (9) |
| Female, n (%) | 4401 (64.7) | 2299 (70.8) | 1401 (60.0) | 1206 (75.6) | 1551 (68.5) |
| Education, n (%)  primary school diploma  secondary school diploma  high school diploma  bachelor's degree  master's degree or single cycle degree  other higher education | 14 (0.2)  679 (10)  2972 (43.7)  840 (12.4)  1865 (27.4)  430 (6.3) | 6 (0.18)  311 (9.5)  1414 (43.5)  414 (12.7)  880 (27.1)  220 (6.8) | 3 (0.1)  167 (7.1)  928 (31.2)  352 (15.1)  722 (30.9)  164 (7.0) | 2 (0.12)  116 (7.3)  654 (41.9)  283 (17.7)  433 (27.8)  107 (6.9) | 4 (0.17)  261 (11.5)  1042 (46.0)  214 (9.4)  590 (26.0)  154 (6.8) |
| Type of work, n (%)  Administrative / Employee  Craftsman / Maneuver  Police Forces  Freelancer  Factory worker  Healthcare Professional  Restauranteur  Student  Other  Unemployed | 1836 (27)  154 (2.3)  58 (0.9)  879 (12.9)  378 (5.6)  645 (9.5)  103 (1.5)  328 (4.8)  1584 (23.2)  835 (12.3) | 1020 (31.4)  83 (2.5)  32 (0.98)  429 (13.2)  201 (6.2)  311 (9.6)  59 (1.8)  9 (0.28)  731 (22.5)  370 (11.4) | 563 (30.7)  44 (1.9)  15 (0.6)  329 (14.1)  135 (5.8)  242 (10.4)  33 (1.4)  318 (13.6)  430 (18.4)  227 (9.7) | 500 (31.3)  35 (2.2)  15 (0.94)  207 (13)  90 (5.6)  172 (10.8)  26 (1.6)  5 (0.31)  340 (21.3)  205 (12.8) | 680 (30.0)  66 (2.9)  22 (0.9)  2929 (12.9)  154 (6.8)  206 (9.1)  44 (1.9)  5 (0.22)  519 (22.9)  277 (12.2) |
| Number in households; median (IQR)  *n (%)*  one  two  three  four  five  more than five people  Number of family members aged over 65 years, mean (sds)  at least one family member over 65 years, n (%)  *n (%)*  one  two  three  four  more than four people | 4 (1)  572 (8.4)  1481 (21.8)  1909 (28.1)  2125 (31.3)  532 (7.8)  181 (2.7)  0.58 ± 1.08  2079 (30.6)  939 (13.8)  718 (10.6)  174 (2.6)  131 (1.9)  117 (1.7) | 4 (1)  25 (0.77)  139 (4.3)  1242 (38.2)  1421 (43.7)  318 (9.8)  100 (3.1)  0.57 ± 1.22  746 (22.9)  208 (6.4)  235 (7.2)  119 (3.6)  101 (3.1)  83 (2.5) | 2.5 (1)  441 (19.4)  944 /41.5)  346 (15.2)  405 (17.8)  149 (6.2)  0 (0.0)  0.63 ± 1.0  858 (36.7)  1478 (63.3)  431 (18.5)  47 /2.0)  23 (1.0)  29 (24.8) | 4 (1)  3 (0.19)  32 (2)  642 (40.2)  717 (44.9)  148 (9.3)  53 (3.3)  0.63 ± 1.30  383 (24)  92 (5.8)  124 (7.8)  57 (3.6)  58 (3.6)  52 (3.3) | 4 (1)  23 (1)  112 (4.9)  659 (29.1)  1100 (48.6)  284 (12.5)  87 (3.9)  0.53 ± 1.32  498 (21.9)  145 (6.4)  161 (7.1)  78 (3.4)  71 (3.1)  43 (1.9) |

*Supplementary Table 2 –* Characteristics of effective Covid-19 involvement (items 13–18) and perceived impact of the pandemic (item 12).

|  | **Whole sample**  **(6800)** | **Caregivers with children aged <18**  **(n = 3245)** | **Non-caregivers**  **(n = 2336)** | *p°* | **Caregivers with children aged <6 years of age**  **(n = 1595)** | **Caregivers with children aged between 6-18 years of age**  **(n = 2265)** | *p°* | **Caregivers of children <18 years with people aged over 65 among households**  **(746)** | **Caregivers of children <18 years without people aged over 65 among households**  **(2499)** | *p°* | **Non-caregivers with people aged over 65 among households**  **(858)** | **Non-caregivers without people aged over 65 among households**  **(1478)** | *p°* |
| --- | --- | --- | --- | --- | --- | --- | --- | --- | --- | --- | --- | --- | --- |
| Covid-19 positive in family members, *n (%)*  Declared  Preferred not to answer | 172 (2.5)  549 (8.1) | 104 (2.3)  285 (6.4) | 70 (3.0)  266 (11.4) | *0.195*  ***<0.001**** | 46 (2.9)  132 (8.3) | 51 (2.3)  89 (3.9) | *0.257*  *0.797* | 21 (2.8)  59 (7.9) | 54 (2.2)  136 (5.4) | *0.329*  *0.012* | 31 (3.6)  109 (12.7) | 37 (2.5)  155 (10.5) | *0.124*  *0.102* |
| Hospitalization for Covid-19 among family members or close friends, *n (%)*  Declared  Preferred not to answer | 1025 (15.1)  34 (0.5) | 678 (15.2)  20 (0.4) | 347 (14.9)  14 (0.6) | ***<0.001****  *0.492* | 253 (15.9)  10 (0.6) | 357 (14.4)  12 (0.5) | *0.968*  *0.857* | 112 (15.0)  6 (0.8) | 391 (15.6)  10 (0.4) | *0.176*  *0.174* | 117 /12,69  8 (0.9) | 230 (15,6)  6 (0.4) | *0.207*  *0.112* |
| Death related to Covid-19 among family members, *n (%)* | 73 (1.1) | 46 (1.0) | 27 (1.2) | *0.056* | 11 (0.7) | 28 (1.2) | *0133* | 12 (1.6) | 21 (0.8) | *0.066* | 15 (1,7) | 12 (0.8) | *0.041* |
| Death related to Covid-19 among close friends, *n (%)* | 567 (8.3) | 404 (9.1) | 163 (7.0) | ***<0.001**** | 138 (8.7) | 215 (9.5) | *0.404* | 76 (10.2) | 227 (9.1) | *0.363* | 67 (7.3) | 100 (6.8) | *0.345* |
| Family members with need for regular care, *n (%)* | 1654 (24.3) | 1082 (24.2) | 575 (24.5) | ***<0.001**** | 418 (26.2) | 576 (25.4) | *0.613* | 263 (35.3) | 556 (22.2) | ***<0.001**** | 263 (30.7) | 309 (20.9) | ***<0.001**** |
| Subjective definition of Covid-19 pandemic on the global population  (Likert scale from 0, not important at all, to 10, extremely important) | 8.91 ± 1.49 | 9.03 ± 1.40 | 8.74 ± 1.51 | ***<0.001**** | 9.04 ± 1.35 | 9.03 ± 1.43 | *0.44* | 9.06 ± 1.49 | 9.02 ±1.37 | *0.493* | 8.8 ±1.48 | 8.7 ± 1.52 | *0.121* |

°statistical significance between the different groups. was calculated using the chi-square test for categorical variables and unpaired t test for continuous variables.

*Supplementary Table 3* – Adult answers about sleep changes during the early quarantine, which constituted the SleepScore (items 25–30).

|  | **Whole sample**  **(6800)** | **Caregivers with children aged <18**  **(n = 3245)** | **Non-caregivers**  **(n = 2336)** | *p°* | **Caregivers with children aged <6 years of age**  **(n = 1595)** | **Caregivers with children aged between 6-18 years of age**  **(n = 2265)** | *p°* | **Caregivers of children <18 years with people aged over 65 among households**  **(746)** | **Caregivers of children <18 years without people aged over 65 among households**  **(2499)** | *p°* | **Non-caregivers with people aged over 65 among households**  **(858)** | **Non-caregivers without people aged over 65 among households**  **(1478)** | *p°* |
| --- | --- | --- | --- | --- | --- | --- | --- | --- | --- | --- | --- | --- | --- |
| Hours of sleep, *n (%)*  Decreased  Increased  Remained the same | 2048 (30.1)  1714 (25.2)  3037 (44.7) | 1150 (35.4)  697 (21.5)  1398 (43.1) | 557 (23.9)  768 (32.9)  1010 (43.3) | ***<0.001****  ***<0.001****  *0.013* | 611 (38.3)  301 (18.9)  683 (42.8) | 788 (34.8)  532 (23,5)  945 (41,7) | *0.027*  ***<0.001****  *0.517* | 289 (38.7)  159 (21.3)  298 (40.0) | 861 (28.5)  538 (21.5)  1100 (44.0) | *0.031*  *0.704*  *0.016* | 218 (25.4)  276 (32.1)  364 (42.5) | 339 (23,0)  492 (33.3)  647 (43.7) | *0.176*  *0.578*  *0.525* |
| Sleeping less than 5 hours, *n (%)* | 365 (5.4) | 193 (5.9) | 97 (4.1) | ***0****.002* | 101 (6.3) | 134 (5,9) | *0.642* | 54 (7.2) | 39 (1.5) | ***<0.001**** | 139 (16.2) | 57 (3.8) | ***<0.001**** |
| Likert scale about difficulties in falling asleep | 4.63 ± 2.69 | 4.70 ± 2.72 | 6.62 ± 0.26 | ***<0.001**** | 4.79 ± 2.69 | 4.70 ± 2.76 | *0.090* | 4.92 ± 2.76 | 4.453 ± 2.71 | ***<0.001**** | 4.67 ±2.71 | 4.58 ± 2.61 | *0.991* |
| Sleep awakenings, *n (%)* | 4259 (62.6) | 2123 (65.4) | 1051 (55.0) | ***<0.001**** | 1065 (66.8) | 1842 (66.1) | ***<0.001**** | 505 (67.7) | 1618 (64.7) | *0.410* | 483 (56.3) | 801 (54.2) | *0.325* |
| Nightmares, *n (%)* | 1670 (24.6) | 816 (25.1) | 686 (29.4) | *0.002* | 445 (27.9) | 607 (21.8) | ***<0.001**** | 215 (28.8) | 601 (24.0) | *0.018* | 246 (28.7) | 440 (29.8) | 0.574 |
| Likert scale about how much sleep was restorative | 5.84 ± 2.16 | 5.60 ± 2.21 | 6.04 ± 2.08 | ***<0.001**** | 4.69 ± 2.75 | 5.72 ± 2.23 | ***<0.001**** | 4.92 ± 2.75 | 4.63 ± 2.71 | *0.010* | 4.67 ± 2.71 | 4.58 ± 2.61 | *0.428* |

°statistical significance between the different groups. was calculated using the chi-square test for categorical variables and unpaired t test for continuous variables.

*Description.* Supplementary Table 3 reveals sleep trends among the respondents. Note how caregivers reported a significant decrease in sleep hours with more difficulties in falling asleep, night awakenings and lower subjective feelings of restorative sleep (p < 0.0001) in comparison to individuals without dependent children, with caregivers with children <6 years group being more affected. Presence of people over 65 years of age among households was generally associated with decrease in sleep ours and dificultise in falling asleep in the caregivers (p < 0.0001) but not in the non-caregivers.

*Supplementary Table 4 –* Behavioral changes related to Covid-19 and confinement in adults.

|  | **Difficulties in concentrating**  *n (%)* | **Exacerbation of chronic diseases**  *n (%)* | **Somatic symptoms**  *n (%)* | **Mood changes**  *n (%)* | **Fear of contamination**  *n (%)* | **Loneliness**  *n (%)* | **Hopelessness^**  *mean (sds)* | **Need for help**  *n (%)* |
| --- | --- | --- | --- | --- | --- | --- | --- | --- |
| **Whole sample**  (6800) | 3762 (55.3) | 3564 (52.4) | 2609 (38.4) | 3889 (57.2) | 4491 (66.1) | 3285 (48.3) | 0.58 ± 0.49 | 1022 (15.0) |
| **Caregivers with children aged <18**  **(n = 3245)** | 1959 (60.4) | 1803 (55.6) | 1353 (41.7) | 2035 (62.7) | 2359 (72.7) | 1494 (46.0) | 0.57 ± 0.50 | 537 (16.5) |
| **Non-caregivers**  **(n = 2336)** | 1244 (53.3) | 1182 (50.6) | 872 (37.3) | 1352 (57.9) | 1401 (60.0) | 1284 (55.0) | 0.51 ± 0.21 | 370 (15.9) |
| *p°* | *0.002* | ***<0.001**** | ***<0.001**** | ***<0.001**** | ***<0.001**** | ***<0.001**** | ***<0.001**** | 0.502 |
| **Caregivers with children aged <6 years of age**  **(n = 1595)** | 1009 (63.3) | 929 (58.2) | 656 (41.1) | **1111 (69.7)** | 1211 (75.9) | **803 (50.3)** | **0.60 ± 0.49** | 281 (17.6) |
| **Caregivers with children aged between 6-18 years of age**  **(n = 2265)** | 1355 (59.8) | 1238 (54.7) | 954 (42.1) | 1370 (60.5) | 1624 (71.7) | 995 (43.9) | 0.54 ± 0.50 | 358 (15.8) |
| *p°* | *0.034* | *0.029* | *0.732* | ***<0.001**** | *0.003* | ***<0.001**** | ***<0.001*** | *0.147* |
| **Caregivers of children <18 years with people aged over 65 among households**  **(746)** | 475 (63.7) | 458 (61.4) | 343 (46.0) | 484 (64.9) | 554 (74.3) | 354 (47.5) | 0.54 ± 0.21 | 140 (18.8) |
| **Caregivers of children <18 years without people aged over 65 among households**  **(2449)** | 1484 (59.4) | 1345 (53,8) | 1010 (40.4) | 1551 (62.1) | 1805 (72.2) | 1140 (45.6) | 0.54 ± 0.22 | 397 (15.9) |
| *p°* | *0.130* | ***0.001*** | *0.021* | *0.441* | *0.775* | *0.665* | *0.260* | *0.102* |
| **Non-caregivers with people aged over 65 among households**  **(858)** | 456 (53,1) | 445 (51,9) | 315 (36.7) | 517 (60.3) | 524 (61.1) | 489 (57.0) | 0.55 ± 0.22 | 132 (15.4) |
| **Non-caregivers without people aged over 65 among households**  **(1478)** | 788 (53.4) | 737 (49.9) | 557 (37.7) | 835 (56.5) | 877 (59.4) | 795 (53.9) | 0.54 ± 0.21 | 238 (16.1) |
| *p°* | *0.973* | *0.351* | *0.639* | *0..075* | 0.409 | 0.133 | *0.275* | *0.733* |

^Hoplessness index is calculated by converting the answer to item 72 (hoplessness) of the Likert scale with the formula: (10-hopelessness)/10

°statistical significance between the different groups. was calculated using the chi-square test for categorical variables and unpaired t test for continuous variables.

| **Age group** | **PsW score** | **CoVidStress score** | **Sleep score** |
| --- | --- | --- | --- |
| **18–28 years** | 1.43 | 3.51 | 2.42 |
| **28–38 years** | 1.41 | 3.45 | 2.67 |
| **38–48 years** | 1.38 | 3.37 | 2.73 |
| **48–58 years** | 1.29 | 2.88 | 2.54 |
| **58–68 years** | 1.21 | 2.54 | 2.4o |
| **68–78 years** | 1.06 | 2.19 | 2.40 |
| **78–88 years** | 0.80 | 1.97 | 2.25 |

*Supplementary Table 5* – Trends in the average composite scores of PsW, CovidStress, and Sleep divided as per age in the sub-sample of adults with dependent children

*Supplementary Table 6 –* Spearman’s correlations among the main behavioral changes in dependents and disturbances of caregiver respondents.

|  | **Caregivers with children aged <6 years of age**  **(n = 1595)** | | | |
| --- | --- | --- | --- | --- |
|  | **PsW score** | **CoVidStress score** | **SubUse score** | **SleepScore** |
| regression | 0.05° | 0.04 | 0.06° | 0.05° |
| enuresis | 0.01 | 0.03 | 0.02 | 0.00 |
| encopresis | 0.01 | 0.02 | −0.00 | 0.03 |
| difficulties in falling asleep | 0.06°° | 0.13* | 0.00 | 0.16* |
| nocturnal awakenings | 0.11* | 0.19* | 0.03 | 0.24* |
| refusal to eat | 0.07+ | 0.09+ | 0.10 * | 0.06+ |
| inconsolable crying | 0.09* | 0.12* | 0.01 | 0.09+ |
| increased irritability | 0.12* | 0.24* | 0.05° | 0.17* |
| restlessness | 0.12* | 0.11* | 0.01 | 0.11* |
| fear of the dark | 0.08+ | 0.10* | 0.03 | 0.08+ |
| crying at the separation from the caregiver | 0.98* | 0.05 | 0.01 | 0.09* |
|  | **Caregivers with children aged between 6-18 years of age**  **(n = 2265)** | | | |
| less cooperation in housekeeping | 0.11* | 0.20* | 0.11* | 0.11* |
| marked need for order and cleanliness | 0.08* | 0.09* | 0.05° | 0.09* |
| use of social media without the purpose of communicating with peers | 0.10* | 0.12* | 0.07+ | 0.07+ |
| difficulty in falling asleep before 11.00 pm | 0.13* | 0.21* | 0.07+ | 0.13* |
| greater difficulty in waking up in the morning | 0.15* | 0.18* | 0.08* | 0.12* |
| crying for no reason | 0.09* | 0.18* | 0.05° | 0.13* |
| feeling of shortness of breath | 0.19* | 0.28* | 0.09* | 0.17* |
| fishbone sensation in the mouth | 0.15* | 0.28* | 0.05° | 0.17* |

° p < 0.05, °° p < 0.005, + p < 0.001, * p < 0.001*

*Supplementary Chart 1 –* Participants to the survey

71 forms were excluded for the inconsistency of responses

6871 subjects completed the survey

6800 subjects were included in the study

4464 subjects with offspring

2336 subjects with no offspring among households

**3245 caregivers with children under 18 years**

1219 subjects with offspring aged over 18 years

2265 subjects with at least one child between 6 and 18 years of age

1595 subjects with at least one child younger than 6 years of age
